# Amelioration of alcohol-induced acute liver injury in C57BL/6 mice by a mixture of TCM phytochemicals and probiotics with antioxidative and anti-inflammatory effects

**DOI:** 10.3389/fnut.2023.1144589

**Published:** 2023-03-07

**Authors:** Zhiguo Li, Xuexun Fang, Xin Hu, Congcong Li, Youzhong Wan, Dahai Yu

**Affiliations:** ^1^China-Japan Union Hospital of Jilin University, Jilin University, Changchun, China; ^2^Key Laboratory for Molecular Enzymology and Engineering of Ministry of Education, School of Life Sciences, Jilin University, Changchun, China

**Keywords:** TCM, phytochemicals, liver injury, probiotics, anti-oxidation, anti-inflammation

## Abstract

**Background:**

There are many causes of acute liver injury (ALI), such as alcohol, drugs, infection, and toxic materials, which have caused major health problems around the world. Among these causes, alcohol consumption induced liver injury is a common alcoholic liver disease, which can further lead to liver failure even liver cancer. A number of traditional Chinese medicine (TCM) and TCM derived compounds have been used in treating the liver-associated diseases and combination use of probiotics with TCM phytochemicals has attracted interests for enhanced biological effects.

**Methods:**

This study investigated the hepatoprotective effect of TCM-probiotics complex (TCMPC) and its underlying mechanism for the treatment of ALI in mice. The TCMPC is composed of TCM phytochemicals puerarin, curcumin, ginsenosides, and 5 *lactobacteria* strains. We first established a mouse model of alcohol-induced ALI, then the therapeutic effects of TCMPC on alcohol-induced ALI were monitored. A series of measurements have been performed on antioxidation, anti-inflammation, and lipid metabolism regulation.

**Results:**

The results showed that TCMPC can reduce the level of liver injury biomarkers and regulate oxidative stress. Histopathological results indicated that TCMPC could ameliorate ALI in mice. In addition, it can also significantly reduce the production of inflammatory cytokines caused by ALI.

**Conclusion:**

Our research has proved the therapeutic effect of TCMPC on alcohol-induced ALI. The potential mechanism of hepatoprotective effects of TCMPC may be related to its antioxidative and anti-inflammatory effects. Our research might provide a new way for liver disease treatment.

## 1. Introduction

Alcohol abuse has been recognized as a major cause of liver injury, including acute and chronic liver injury (1). In the region with the highest alcohol consumption (Europe), there are about 287,000 premature deaths related to liver disease each year, of which about 40% are caused by alcohol (2). Alcohol-induced acute liver injury (ALI) is a common alcoholic liver disease, which refers to sudden liver damage caused by heavy drinking, and is one of the common causes of liver failure and even liver cancer (3).

Acetaldehyde is the major toxic metabolite of ethanol. One of the targets of acetaldehyde is mitochondria, and mitochondrial damage induces over accumulation of reactive oxygen species (ROS) and the reduction of antioxidant activities (4). Under physiological conditions, ROS are efficiently eliminated by antioxidant defense systems including superoxide dismutase (SOD) and glutathione peroxidase (GSH-Px) (5). However, the hyper-levels of ROS, acting as a second messenger, enhance the transportation and activation of nuclear factor kappa B (NF-κB), a reduction/oxidation (redox)-sensitive factor, from the cytoplasm into the nucleus (6). The activation of NF-κB inflammatory pathway accelerates the release of inflammatory cytokines including tumor necrosis factor-α (TNF-α), interleukin-1-β (IL-1β), and interleukin-6 (IL-6), exacerbating hepatic inflammation and systemic injury (7, 8). So the inhibition of NF-κB activation and cytokine synthesis is a potential mechanism for the treatment of alcohol-induced liver injury.

Increasing studies have demonstrated a two-way communication between the gut and liver (9). Alcohol can cause intestinal flora change, intestinal epithelial cell barrier dysfunction, intestinal bacterial metabolite translocation, and endotoxemia, suggesting that intestinal flora plays an important role in alcohol-induced liver damage (10). Inhibiting oxidative stress, preventing inflammation, and restoring intestinal homeostasis are promising therapeutic approaches to treat ALI.

Common ALI treatments include antioxidant drugs, anti-fibrosis drugs, anti-inflammatory drugs, glucocorticoids, and cell membrane protective agents (11). Some of these drugs may also have adverse effects on patients such as diarrhea, allergy, gastric irritation, and kidney damage. In order to avoid the metabolic burden of organs, these drugs are not suitable for long-term use, and long-term use may produce drug resistance, which is not conducive to or even aggravate the condition of alcoholic liver disease (12). At present, some emerging ALI treatment strategies include microecological therapy based on intestinal-liver axis, hepatocyte regeneration technology, and targeted pathogenic molecular therapy (13). Increasing studies have shown that the intestinal-liver axis is related to the occurrence and development of alcoholic liver disease. The intestinal-liver axis refers to the bidirectional relationship between the gut with its microbiota and the liver, which is generally established by the portal vein and regulated by diet, genetics, and environmental factors (14). Bile acids produced in the liver regulate microbiota composition and gut barrier function, and gut products regulate bile acid synthesis and glucose and lipid metabolism in the liver (15). The intestinal barrier can limit the systemic spread of microorganisms and toxins, while allowing nutrients to enter the portal circulation and reach the liver. Alcohol has been shown to alter gut microbiome composition and impair gut integrity and barrier function in addition to its direct toxicity to hepatocytes. Alcohol can cause changes in the expression of tight junction proteins in the intestine, such as zonula occludens-1 (ZO-1), thereby increasing intestinal permeability (16). Disruption of the gut microbiota and increased intestinal permeability can lead to the influx of endotoxins such as lipopolysaccharide (LPS) into the portal circulation, which activate toll-like receptor 4 (TLR4) to promote inflammation in alcoholic liver disease (17).

Traditional Chinese medicine (TCM) has been used for the treatment of diseases, and it is still regarded as an important source of therapeutic drugs (18). Many studies have now demonstrated the therapeutic effects of TCM in cancer, cardiovascular disease, inflammation, and liver disease (19–22). Some low-toxic Chinese herbs, such as Salvia miltiorrhiza, licorice, Radix Puerariae, and Panax ginseng C. A. Meyer, contain a variety of active ingredients such as terpenoids, flavonoids, alkaloids, phenols, polysaccharides, and other active ingredients, which can play a strong role in anti-inflammation and antioxidation, improve the immune function of the body, and regulate intestinal flora (23–26). Puerarin is a bioactive flavone isolated from Puerariae Lobatae Radix (gegen), an herbal TCM used for many pathological conditions; Curcumin is the main ingredient of Curcuma longa L., commonly known as turmeric (jianghuang), a plant of high medicinal values and widely used especially in Southeast Asia; Ginsenosides are saponins, which are the major pharmacologically active components of Panax Ginseng (renshen). Total ginsenosides are isolated and purified from ginseng roots, and are a mixture of ginsenosides such as Rg1, Re, Rb1, Rc, and Rd. These TCM phytochemicals have shown many biological effects such as antioxidant, anti-inflammatory, antidiabetic, and anticancer. Puerarin is widely used as a dietary supplement and has shown good effects in the treatment of obesity, diabetes, cardiovascular, cerebrovascular diseases, and inflammatory diseases (27). Curcumin has a variety of biopharmacological effects, including antioxidation, anticancer, liver protection, immune regulation, and hypoglycemia *in vitro* or *in vivo* (28). The pharmacological studies of ginsenosides have focused on their anticancer, antioxidant, and anti-inflammatory activities (29). Zhao et al. found that Rg1 has a positive role in the treatment of CCI4-induced ALI mouse model, which may be related to NF-κB /NLRP3 inflammasome signaling pathway (30). In traditional Chinese medicinal practice, a number of herbs with different properties are mixed for optimal therapeutic effects.

Based on the intestinal-liver relationship, modulation of the gut microbiota, including probiotics, fecal microbiota transplantation, and antibiotics, has been investigated in the treatment of alcoholic liver disease with varying degrees of success. Probiotics are biologically active bacteria that also have a variety of beneficial effects, such as regulating immune responses, maintaining intestinal barrier homeostasis, promoting nutrient absorption, and improving intestinal flora imbalance (31, 32). Previous studies have demonstrated the therapeutic effects of probiotics on cancer, hypertension, diabetes, and alcoholic/non-alcoholic fatty liver disease (33–35). Li et al. showed that a mixture of *Lactobacillus plantarum* KLDS1.0344 and *Lactobacillus acidophilus* KLDS1.0901 can improve intestinal epithelial cell permeability and reduce serum LPS levels, thereby inhibiting alcohol-induced liver inflammation (12). Using a mouse model, Christoph et al. found that *A. muciniphila* can promote gut barrier integrity and improve experimental alcoholic liver disease (36). TCM-probiotics complex (TCMPC) is composed of TCM phytochemicals and a variety of probiotics. Here, we used puerarin, curcumin, total ginsenosides, and 5 *lactobacteria* strains to formulate TCMPC to treat ALI in a mouse model. The TCMPC preparation has the advantage of both probiotics and TCM compounds. As TCM compounds may benefit the growth of probiotics, and probiotics can also promote the absorption and utilization of TCM compounds (37).

This study focused on the liver damaging response and hepatic pathology alteration caused by alcohol-induced ALI. Further, anti-inflammatory and antioxidant effects of TCMPC preparation on the treatment of alcohol-induced ALI were monitored.

## 2. Materials and methods

### 2.1. Preparation of TCMPC

TCMPC is the mixture of probiotics and TCM phytochemicals. The ingredients of TCMPC are shown in Table 1. Puerarin (purity ≥98% by HPLC), curcumin (purity ≥98% by HPLC), and total ginsenosides (purity ≥80% by UV spectrometry) were purchased from Shanghai Yuanye Biological Technology Co., Ltd (Shanghai, China). The above drug quality inspection data are shown in Supplementary Figures S1–S3. *Lactobacillus animalis*-BA12*, Lactobacillus bulgaricus*-LB42*, Lactobacillus paracasei*-LC86*, Lactobacillus casei*-LC89, and *Lactobacillus plantarum*-LP90 were all purchased from Wecare Probiotics Co., Ltd (Suzhou, China).

**Table 1:** The main ingredients of TCMPC.

TCM compounds	Active ingredient /Formula	Chemical structures	Solubility (In water)	TCM content (w/w)	Probiotics strain	Probiotics content (CFU/g)
Puerarin	C_21_H_20_O_9_	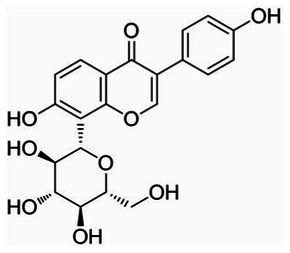	Soluble (Heating)	40	*Lactobacillus animalis-BA12*	2 × 10^12^
Curcumin	C_21_H_20_O_6_	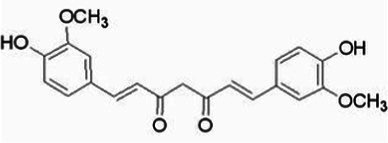	Insoluble	20	*Lactobacillus bulgaricus-LB42*	2 × 10^12^
Total ginsenosides	Rg1 (C_42_H_72_O_14_)	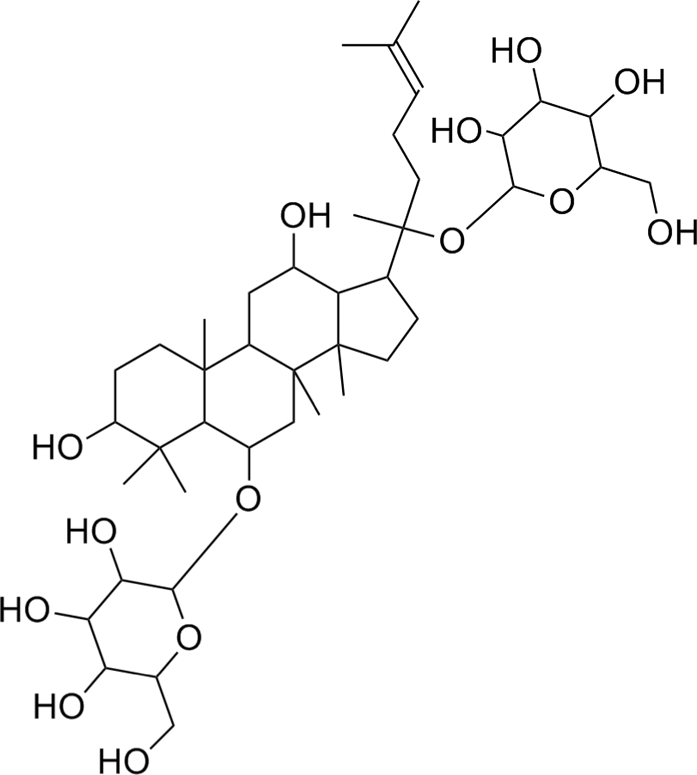	Soluble	1	*Lactobacillus paracasei-LC86*	2 × 10^12^
	Re (C_48_H_82_O_18_)	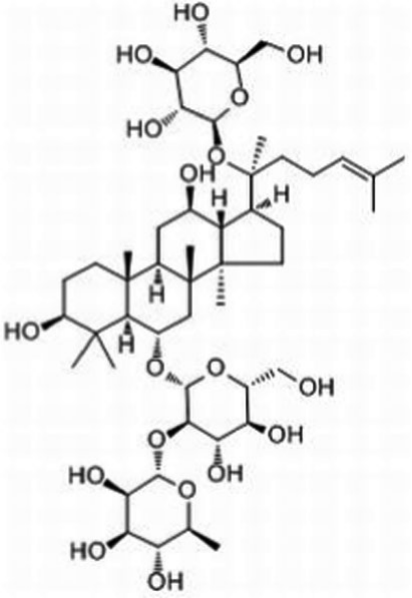			*Lactobacillus casei-LC89*	2 × 10^12^
	Rb1 (C_54_H_92_O_23_)	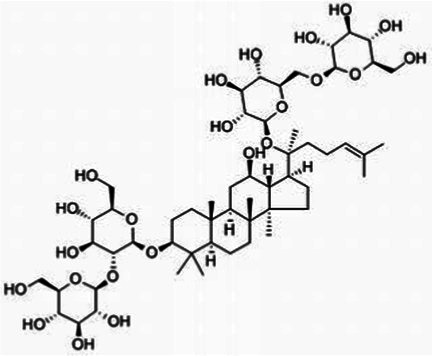			*Lactobacillus plantarum-LP90*	2 × 10^12^
	…	…				

### 2.2. Animal models and treatment

**Figure 1:**
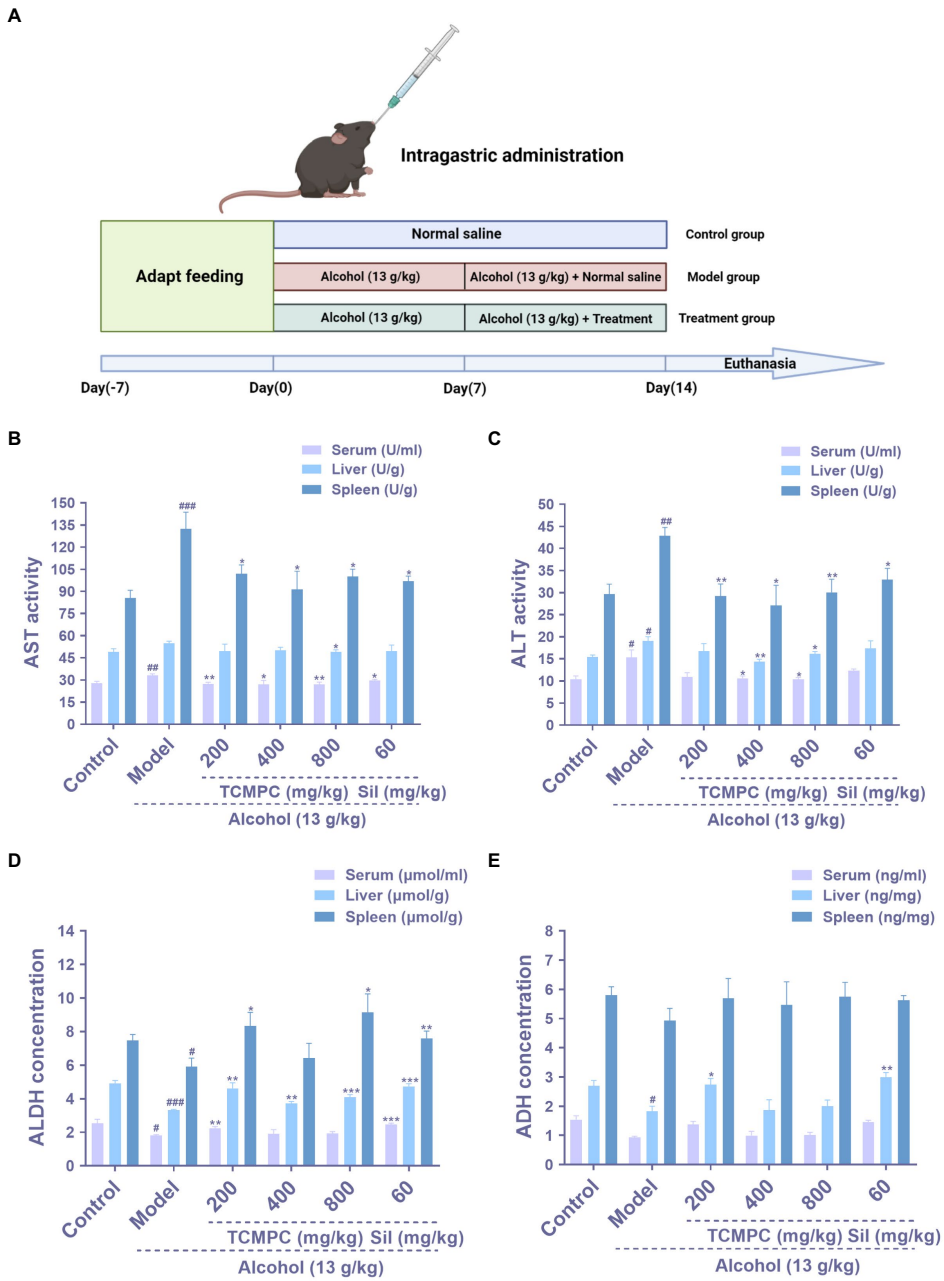
The hepatoprotective effects by TCMPC in mice with alcohol-induced ALI. Schematic diagram of the administration of alcohol-induced ALI mice in the control group, model group, and treatment groups after 1 week of adaptive feeding **(A)**, The effects of TCMPC on AST levels of serum, liver, and spleen, respectively, in alcohol-treated mice **(B)**, The effects of TCMPC on ALT levels of serum, liver, and spleen, respectively **(C)**, The effects of TCMPC on ALDH levels of serum, liver, and spleen, respectively **(D)**, and The effects of TCMPC on ADH levels of serum, liver, and spleen, respectively **(E)**. Data are expressed as mean ± SD, *n* = 10. ^#^*p* < 0.05, ^##^*p* < 0.01, and ^###^*p* < 0.001, versus control group. ^*^p < 0.05, ^**^p < 0.01, and ^***^p < 0.001, versus model group. AST, aspartate aminotransferase; ALT, alanine aminotransferase; ALDH, aldehyde dehydrogenase; ADH, antidiuretic hormone.

The methodology for establishing the mice model with alcohol-induced ALI was modified according to previous studies (38). The mice were randomly divided into six groups (n = 10/group): control group, model group, positive control group, and TCMPC treatment group that conclude three group: high dose group (800 mg/kg), medium dose group (400 mg/kg), and low dose group (200 mg/kg). The mice in the control group were treated by intragastric administration of normal saline at 0.1 ml/10 g twice a day at 9:00 am and 4:00 pm for 14 days. The mice in the other groups were gavaged with the white spirit (56°, Beijing Shunxin Agricultural Co. Ltd., China) at 9:00 am once every day and the dosage of it is 13 g/kg. And after 7 days, every day at 4:00 pm, the mice in the model group were gavaged with normal saline, the mice in the positive control group were intragastric administrated with silymarin (Sil) (Tianjin Tasly Sants Pharmaceutical Co. Ltd., China), a clinical used liver protectant, which can reduce oxidative stress in patients with alcoholic liver disease and prevent the lipid peroxidation. The mice in other treatment group were given TCMPC at the dose of 200 mg/kg, 400 mg/kg and 800 mg/kg, respectively. Before administration, TCMPC was dissolved in 1.5% (w/v) sodium carboxymethyl cellulose solution. The above specific operation flow is shown in Figure 1A. The body weight of mice was monitored every day for 14 days. Following the last administration, all mice were fasted overnight before taking blood from their eyeballs, and all the mice were euthanized by CO_2_. After dissection, the various organs of the mice were rapidly removed and frozen at −80°C for further analysis. At the same time, part of the liver tissue was collected and put into formalin buffer. Animal experiment and grouping.

### 2.3. Biochemical indicators detection

The mouse blood was collected and allowed to coagulate naturally at room temperature for 20–30 min, and then centrifuged at 4°C for 20 min (3,000 rpm) to obtain mouse serum. Take an appropriate amount of organ tissue and put it in normal saline, grind it on ice, and then centrifuge it at 4°C for 10 min (3,500 rpm) to obtain tissue fluid. The levels of aspartate aminotransferase (AST; CK-E90386M), alanine aminotransferase (ALT; CK-E90314M), antidiuretic hormone (ADH; CK-E92648M), aldehyde dehydrogenase (ALDH; CK-E92649M), nitric oxide (NO; CK-E20293M), reactive oxygen species (ROS; CK-E91516M), superoxide dismutase (SOD; CK-E20348M), catalase (CAT; CK-E92636M), glutathione peroxidase (GSH-Px; CK-E92669M), and malondialdehyde in (MDA; CK-E20347M), high-density lipoprotein (HDL; CK-E91912M), triglyceride (TG; CK-E91830M), and total cholesterol (TC; CK-E91839M) were detected using enzyme-linked immunosorbent assay (ELISA) kits purchased from the Shanghai Yuanye Biological Technology Co., Ltd (Shanghai, China) according to the operating instructions.

### 2.4. Histopathological analysis

Part of the liver was excised and fixed in 10% (v/v) neutral formalin buffer. Afterward, the fixed tissues were dehydrated with gradient ethanol (70%, 80%, 90%, 95%, and 100%), and then samples were transparent twice in xylene and embedded in paraffin. The paraffin samples were sliced into 5 μm thickness, and then stained with hematoxylin and eosin (H & E). After dehydration, the pathological sections were observed under light microscope (200×, Olympus, Japan) and photographed.

### 2.5. Statistical analysis

All statistical analyzes were performed using SPSS 23.0 software (IBM Corporation, Armonk, NY, United States). Data were presented as means ± standard error of the mean (S.E.M.). Differences were tested by one-way analysis of variance (ANOVA). *p* values of <0.05 were considered statistically significant.

## 3. Results

### 3.1. The TCMPC exhibited hepatoprotective effects in mice with alcohol-induced ALI

AST and ALT are two hepatic-specific enzymes that reflect the degree of acute liver damage and they are frequently elevated after excessive alcohol intake (39). When liver cells are damaged, cell membrane permeability increases and AST and ALT are released into the blood, increasing serum transaminase content, which is the essential enzyme in metabolic processes (40). In serum, the activities of AST and ALT of mice in the control group were 27.93 U/mL and 10.41 U/ml, those in the model group were 33.18 U/mL and 15.37 U/mL, and those in the TCMPC high dose group were 27.02 U/mL and 10.39 U/mL. Compared with the control group, the activities of AST and ALT in the model group was significantly higher, while TCMPC treatment significantly decreased the activities of AST and ALT (*p* < 0.05; Figures 1B,C). Similar results were also observed in liver and spleen tissue, where the activities of AST, ALT were increased by alcohol treatment, whereas TCMPC treatment prevented these increases (*p* < 0.05; Figures 1B,C). Overall, TCMPC significantly reduced the activities of AST and ALT in all cases at different dosages (200, 400, and 800 mg/kg).

Excessive alcohol consumption can result in over accumulation of acetaldehyde in the liver, which promotes the formation of protein adducts through reactions with various macromolecules in the body, leading to functional impairment of key proteins. ADH and ALDH are two key enzymes responsible for ethanol/acetaldehyde metabolism, and they are involved in the susceptibility to alcoholism and alcohol-related liver damage and diseases (41). Significant decrease (*p* < 0.05; Figures 1D,E) of the levels of ALDH and ADH was observed in model group compared with that of control group. The levels of ALDH and ADH were 4.91 ng/mg and 2.7 ng/mg in liver of mice in the control group and 3.32 ng/mg and 1.83 ng/mg in the model group. Comparatively, TCMPC treatments significantly ameliorated the changes in the levels of ALDH and ADH in alcohol-injured mice (Figures 1D,E) to 4.09 ng/mg and 2.0 ng/mg in TCMPC high dose group.

### 3.2. TCMPC enhanced antioxidant capacity in alcohol-induced ALI mice

**Figure 2:**
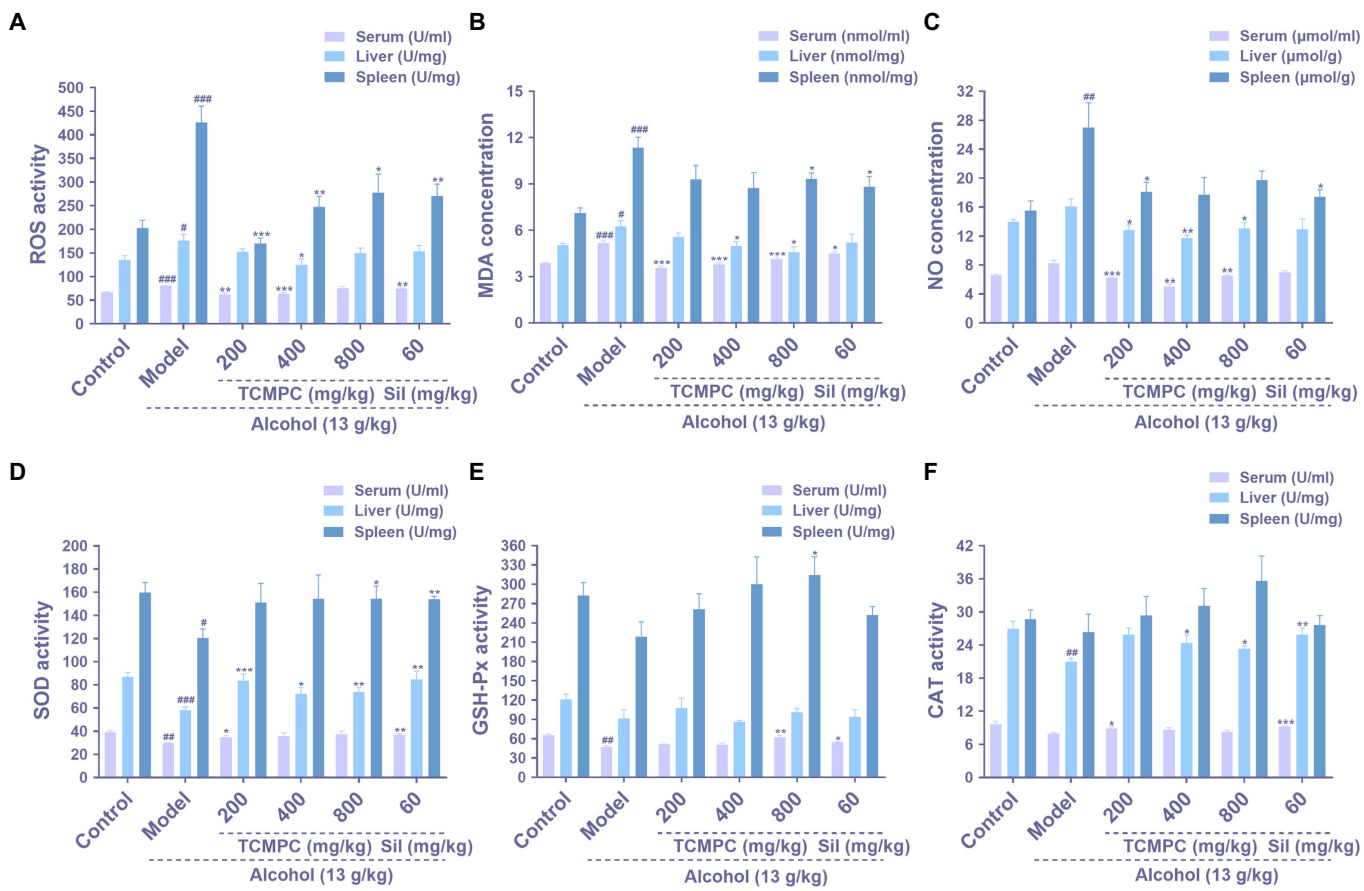
Antioxidative effects of TCMPC on alcohol-induced ALI mice. The levels of ROS **(A)**, MDA **(B)**, NO **(C)**, SOD **(D)**, GSH-Px **(E)**, and CAT **(F)** in serum, liver, and spleen were detected by ELISA kit. Data are expressed as mean ± SD, *n* = 10. ^#^*p* < 0.05, ^##^*p* < 0.01, and ^###^*p* < 0.001, versus control group. ^*^p < 0.05, ^**^p < 0.01, and ^***^p < 0.001, versus model group.

Oxidative stress is one of the most important processes in the pathogenesis of ALI. ROS is a natural by-product of normal metabolism of oxygen, but during environmental stress, the level of ROS increases sharply, which may cause serious damage to cell structure known as oxidative stress (42). The interaction between NO and ROS will form reactive nitrogen species (RNS), and the excessive accumulation of RNS will also cause cell and tissue damage (43). MDA is a product of lipid peroxidation, and its overexpression indicates oxidative stress in the body (44). The ROS, MDA, and NO levels of serum, liver, and spleen were significantly increased (*p* < 0.05; Figures 2A–C) in model group compared to the control group. The levels of ROS, MDA, and NO in serum of mice in the control group were 66.52 U/mL, 3.85 nmol/mL, and 6.54 μmol/mL; 79.93 U/mL, 5.17 nmol/mL, and 8.24 μmol/mL in the model group; 75.23 U/mL, 4.13 nmol/mL, and 6.49 μmol/mL in the TCMPC high dose group, respectively. In general, administration of TCMPC reduced the levels of these indicators and showed a very significant difference (*p* < 0.05).

Enzymatic antioxidant system is essential for cellular response in order to deal with oxidative stress under physiological condition. Antioxidant enzyme such as CAT, SOD, and GSH-Px are affected and used as indexes to evaluate the level of oxidative stress, as SOD can convert superoxide into H_2_O_2_, and CAT can convert H_2_O_2_ into H_2_O (45, 46). Alcohol significantly destroys the antioxidant defense enzyme activity in the body. The activities of SOD, GSH-Px, and CAT in serum of mice in the control group were 38.93 U/mL, 65.19 U/mL, and 9.59 U/mL; 29.79 U/mL, 47.33 U/mL, and 7.94 U/mL in the model group; 37.09 U/mL, 62.35 U/mL, and 8.26 U/mL in the TCMPC high dose group. Similar results were also observed in liver and spleen (Figures 2D–F). Our results showed that the enzyme activities of SOD, GSH-Px, and CAT in the model group were significantly decreased compared with the control group, and administration of TCMPC prevented the reduction in the SOD, GSH-Px, and CAT levels in the serum, liver, and spleen of mice with alcohol-induced ALI. And TCMPC displayed even better antioxidative effects than Sil against alcohol-induced ALI in mice in most cases.

### 3.3. Regulation of lipid metabolism and inflammatory cytokines in mice with alcohol-induced ALI

**Figure 3:**
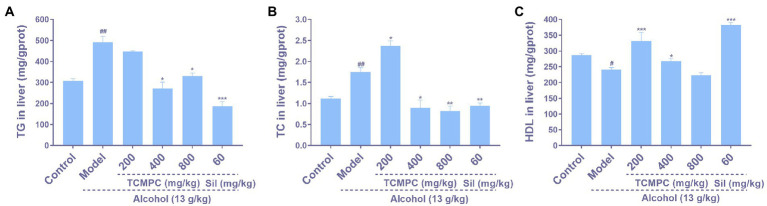
Effect of TCMPC on TG **(A)**, TC **(B)**, and HDL **(C)** levels in mice with alcohol-induced ALI. Data are expressed as mean ± SD, *n* = 10. ^#^*p* < 0.05, ^##^*p* < 0.01, and ^###^*p* < 0.001, versus control group. ^*^p < 0.05, ^**^p < 0.01, and ^***^p < 0.001, versus model group.

Lipid metabolism, especially the levels of TG, TC, and HDL, can serve as a metric indicating the extent of liver injury (47). Alcohol elevated TG and TC levels and lowered HDL levels in the liver of the mice (*p* < 0.05; Figures 3A–C). Compared with model group mice, TCMPC reduced the levels of TG and TC by 44.8% (*p* < 0.01; Figure 3A) and 53.1% (*p* < 0.05; Figure 3B), and enhanced the levels of HDL by 37.9% (*p* < 0.001; Figure 3C). The regulation efficiency of hepatic levels of TG, TC, and HDL by TCMPC were comparable with those of Sil.

**Figure 4:**
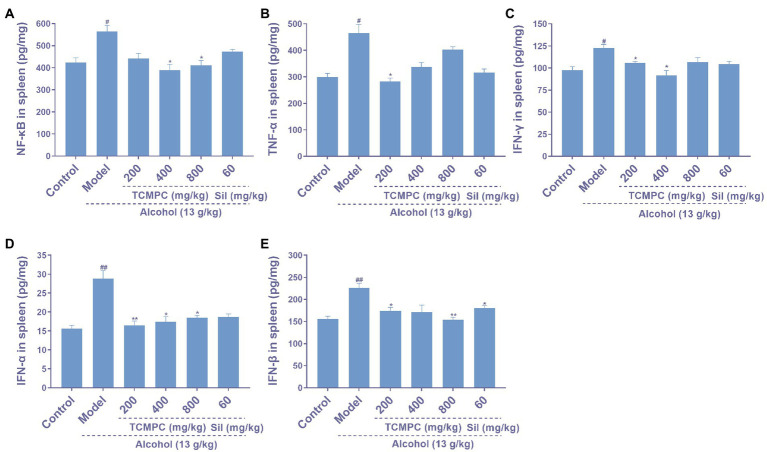
The effects of TCMPC on inflammatory cytokines levels in alcohol-induced ALI mice. The levels of NF-κB **(A)**, TNF-α **(B)**, IFN-γ **(C)**, IFN-α **(D)**, and IFN-β **(E)** in spleen were detected by ELISA kit. Data are expressed as mean ± SD, *n* = 10. ^#^*p* < 0.05, ^##^*p* < 0.01, and ^###^*p* < 0.001, versus control group. ^*^p < 0.05, ^**^p < 0.01, and ^***^p < 0.001, versus model group.

Alcohol also causes the accumulation of inflammatory factors in the liver of mice, leading to the occurrence of hepatitis. For example, NF-κB is a key inflammatory response mediator and can regulates multiple aspects of innate and adaptive immune function (48). The spleen is an important immune organ, and changes in the levels of inflammatory cytokines in the spleen can effectively reflect the inflammatory state of the body (49). NF-κB levels in control and model group were 424.31 pg./mg and 564.93 pg./mg, respectively; TNF-α were 299.46 pg./mg and 465.7 pg./mg; IFN-α were 15.62 pg./mg and 28.85 pg./mg; IFN-β were 155.98 pg./mg and 226.09 pg./mg; IFN-γ were 97.56 pg./mg and 122.66 pg./mg. The levels of NF-κB, TNF-α, IFN-α, IFN-β, and IFN-γ in high dose group mice were 410.82 pg./mg, 402.06 pg./mg, 18.47 pg./mg, 153.94 pg./mg, and 106.91 pg./mg, respectively. Our results showed that the levels of NF-κB, TNF-α, IFN-α, IFN-β, and IFN-γ in spleen were significantly increased (*p* < 0.05; Figure 4) in the model group compared to those of the control group. And TCMPC treatment reduced the levels of these inflammatory factors, showing an anti-inflammatory effect (Figure 4).

### 3.4. Effect of TCMPC on the histopathological changes of liver tissue In mice with alcohol-induced ALI

**Figure 5:**
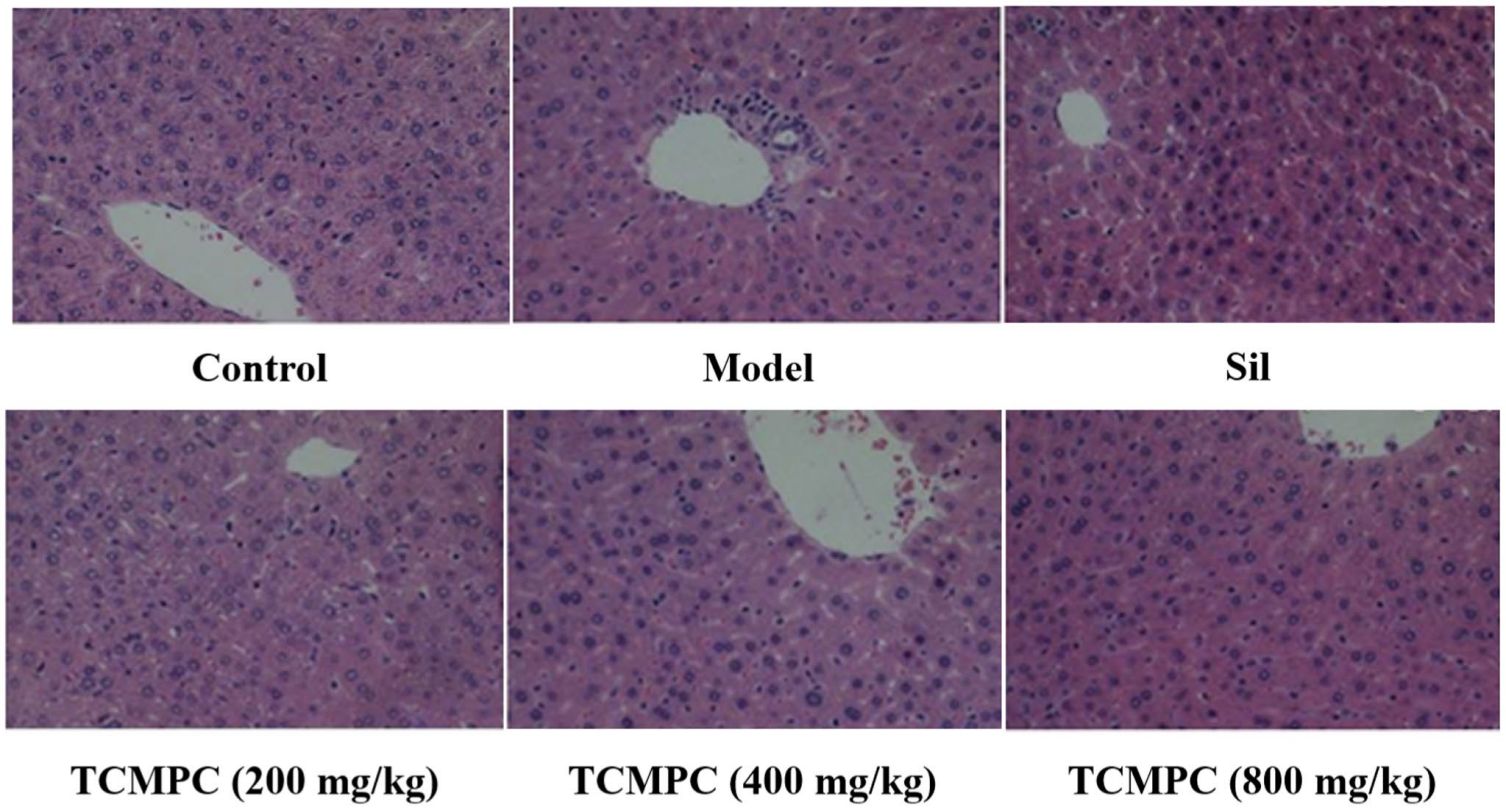
Liver pathology by H&E staining. Representative H&E-stained liver tissue sections are shown at 200x magnification.

Light microscope observation showed that hepatic lobules were intact in normal control group, hepatocytes structure and the morphology were normal; the liver cells were arranged radially and the cells were closely arranged with hepatocyte outline, nucleus clarity, and no necrosis (Figure 5). The hepatic lobule structure was damaged in the model group, as the boundaries were hazy, the arrangement of hepatic cells was disordered, hepatocytes were significantly swollen, and the nucleus was shriveled. There were diffuse fat vacuoles of different sizes in the cytoplasm and extensive infiltration of inflammatory cells. Compared with the model group, the histopathological changes of Sil and TCMPC treatment group of liver injury was significantly alleviated, as the inflammatory cells infiltration and necrosis in the liver of the mice were decreased, slight swelling of some hepatocytes and most of the liver cell structure becomes tight and few inflammatory infiltration areas can be found.

## 4. Discussion

The main cause of liver injury is excessive drinking, including acute and chronic liver injury. Alcohol-induced ALI is a life-threatening disease and has become a public health problem worldwide (50). The metabolic mechanism of alcohol is very complex. Acetaldehyde, as the main toxic metabolite of ethanol, can directly damage mitochondria. At present, more and more evidence showed that there is a close relationship between intestinal microflora and liver injury. Studies have shown that acute exposure to high concentration of ethanol will lead to intestinal microflora imbalance and mucosal damage, which, in turn, increase intestinal permeability and lead to the transfer of endotoxin from the intestinal tract to the liver (51). At the same time, ALI usually causes inflammation and oxidative stress in the body. In recent years, based on anti-inflammatory, antioxidant, and regulation of intestinal microflora, more and more methods of ALI prevention and adjuvant therapy have been proposed (52, 53). Compared with other drugs, TCM has many advantages, including low toxicity, fewer side effects, and less risk of drug resistance (54). It has been reported that Radix Puerariae extract can treat alcoholic liver disease by improving alcohol-induced intestinal barrier dysfunction, and Panax ginseng C. A. Meyer extract can treat non-alcoholic fatty liver disease by regulating intestinal flora (26, 55). Rhubarb extract pretreatment can improve alcohol-induced ALI in mice by improving intestinal homeostasis and restoring intestinal barrier function, especially by increasing the relative abundance of *Akkermansia muciniphila* in cecal contents, which is considered to be a potential probiotic (56). Yang et al. found that puerarin can treat ALI in LPS/D-Gal-induced by increasing the expression of E-box-binding homeobox 2 and inhibiting the activation of NF-κB signal pathway (57). Zhong et al. have shown that curcumin can inhibit oxidative stress-related inflammation through PI3K/AKT and NF-κB related signals, and reduce LPS-induced septicemia and liver injury in mice (58). It has been reported that ginsenoside Rb1 plays a hepatoprotective role in acetaminophen-induced ALI model in mice by regulating the inflammatory response mediated by MAPK and PI3K/Akt signaling pathways (59). A variety of human diseases, including metabolic syndrome, cancer, inflammatory diseases, and infections, are associated with changes in intestinal microbiome. The interaction between intestinal microbiome, intestinal barrier, and liver seems to play a key role in the pathogenesis of alcoholic liver disease, so it is necessary to further explore the intestinal-liver axis in ALI. It is reported that *Lactobacillus rhamnosus* GG culture supernatant can improve the intestinal integrity and liver injury of alcohol-induced ALI mice (60). *Bifidobacterium longum* R0175 and *Bifidobacterium adolescentis* CGMCC 15058 can reduce ALI induced by D-galactosamine, enhance intestinal barrier, and improve intestinal microflora in rats (61). Of course, the improvement of probiotics in ALI needs to be further studied in clinical studies. In this study, we used TCMPC, a mixture of several probiotics and TCM, to treat alcohol-induced ALI mice in order to provide a new method for the treatment of ALI.

Excessive drinking usually leads to the increase of AST and ALT (39). ADH and ALDH are two key enzymes in ethanol metabolism, and their lack of function makes it impossible for ethanol to be metabolized normally, which eventually leads to liver damage (41). TCMPC treatment can significantly reduce the levels of AST and ALT in serum, liver and spleen, increase the levels of ALDH and ADH, and reduce alcohol-induced ALI in mice, this is consistent with the results of Xiao *et al* (62). ALI induces oxidative stress, which increases the level of strong oxidants such as ROS, destroys cellular macromolecules, and leads to hepatocyte damage (63). Antioxidant enzymes such as CAT, SOD, and GSH-Px can be used as indicators to evaluate the level of oxidative stress, and the level of MDA can also be used as an index to evaluate oxidative stress, which is a product of lipid oxidation (46). The levels of ROS, MDA, and NO in serum, liver, and spleen in the model group were significantly higher than those in the control group, and TCMPC could significantly decrease the above-mentioned indexes. Compared with the control group, the activities of SOD, GSH-Px, and CAT in the model group decreased significantly, but TCMPC could prevent this phenomenon to a certain extent. The above results suggest that TCMPC has a protective effect on ALI in mice, which may be achieved by inhibiting oxidative stress and enhancing antioxidant capacity.

The levels of TG, TC and HDL can also be used as indicators of the degree of liver injury. Previous studies have found that alcohol can increase the levels of TG and TC and decrease the level of HDL in the liver of mice (64). Compared with the model group, the serum TG of TCMPC treatment group decreased by 44.8%, TC decreased by 53.1%, and HDL increased by 37.9%. Abnormal cytokine metabolism is another major feature of alcohol-induced ALI. Alcohol leads to the accumulation of inflammatory factors, which leads to the occurrence of hepatitis (12). The levels of pro-inflammatory cytokines NF-κB, TNF-α, IFN-α, IFN-β, and IFN-γ in the model group were significantly higher than those in the control group, which was consistent with previous studies (65). TCMPC can significantly reduce the level of the above inflammatory factors. In addition, alcohol causes liver damage, such as disordered arrangement of hepatocytes, nuclear atrophy, and infiltration of inflammatory cells. Compared with the model group, the liver injury, inflammatory cell infiltration, and cell necrosis in TCMPC treatment group were significantly alleviated.

## 5. Conclusion

In summary, this study shows the therapeutic effect of TCMPC on alcohol-induced ALI, and the potential mechanism may be through anti-inflammation and antioxidation in mice. Compared with the mice in the model group, after oral administration of TCMPC, the levels of ROS, MDA, and NO in ALI mice were significantly decreased, and the levels of CAT, SOD, and GSH-Px were significantly increased, indicating that TCMPC has a strong antioxidant capacity. At the same time, compared with the mice in the model group, pro-inflammatory cytokines such as NF-κB, TNF-α, IFN-α, IFN-β, and IFN-γ were significantly decreased in ALI mice after oral administration of TCMPC, which showed the anti-inflammatory ability of TCMPC. Although TCMPC displayed even better amelioration effects than Sil against alcohol-induced ALI in mice in the majority of cases, it is hard to conclude which one shows better effects based on our present data. Our research provides a new scheme for the treatment of liver disease.

## Data availability statement

The raw data supporting the conclusions of this article will be made available by the authors, without undue reservation.

## Ethics statement

The animal study was reviewed and approved by Jilin University.

## Author contributions

ZL: writing – original draft, methodology, and formal analysis. XF: writing – original draft, investigation, and supervision. XH: resources and writing – review and editing. CL: writing – original draft, methodology, and data curation. YW: project administration, writing – review and editing, and supervision. DY: conceptualization, writing – review and editing, and funding acquisition. All authors contributed to the article and approved the submitted version.

## Funding

This work was supported by Jilin Province Science and Technology Development Project [nos. YDZJ202101ZYTS080, 20200708076YY], and Jilin Provincial Department of Education [no. JJKH20220974CY].

## Conflict of interest

The authors declare that the research was conducted in the absence of any commercial or financial relationships that could be construed as a potential conflict of interest.

The handling editor declared a shared affiliation with the authors at the time of review.

## Publisher’s note

All claims expressed in this article are solely those of the authors and do not necessarily represent those of their affiliated organizations, or those of the publisher, the editors and the reviewers. Any product that may be evaluated in this article, or claim that may be made by its manufacturer, is not guaranteed or endorsed by the publisher.

## Supplementary material

The Supplementary material for this article can be found online at: https://www.frontiersin.org/articles/10.3389/fnut.2023.1144589/full#supplementary-material

## Figures and Tables

**Table:** 

ADH	Antidiuretic hormone
ALT	Alanine aminotransferase
ALDH	Aldehyde dehydrogenase
ALI	Acute liver injury
AST	Aspartate aminotransferase
CAT	Catalase
ELISA	Enzyme-linked immunosorbent assay
GSH-Px	Glutathione peroxidase
HDL	High-density lipoprotein
H & E	Hematoxylin and eosin
IκB	Inhibitor of nuclear factor kappa-B
IFN	Interferon
LPS	Lipopolysaccharide
MDA	Malondialdehyde
NF-κB	Nuclear factor kappa B
NO	Nitric oxide
ROS	Reactive oxygen species
Sil	Silymarin
SOD	Superoxide dismutase
TC	Total cholesterol
TCM	Traditional Chinese medicine
TCMPC	TCM-probiotics complex
TG	Triglyceride
TLR4	Toll-like receptor 4
TNF-α	Tumor necrosis factor-α
ZO-1	Zonula occludens-1
