# Supplementary material for: Amelioration of alcohol-induced acute liver injury in C57BL/6 mice by a mixture of TCM phytochemicals and probiotics with antioxidative and anti-inflammatory effects
Source: Front Nutr. 2023 Mar 7;10:1144589. doi: 10.3389/fnut.2023.1144589 (PMC10027757; doi:10.3389/fnut.2023.1144589)
Supplement: Supplementary file 1 [file Data_Sheet_1.docx]

Amelioration of alcohol-induced acute liver injury in C57BL/6 mice by a mixture of TCM phytochemicals and probiotics with anti-oxidative and anti-inflammatory effects

Zhiguo Li^1†^, Xuexun Fang^2†^, Xin Hu^1^, Congcong Li^2^, Youzhong Wan^1*^ and Dahai Yu^2*^

^1^ China-Japan Union Hospital of Jilin University, Jilin University, Changchun, China 130033, P. R. China

^2^ Key Laboratory for Molecular Enzymology and Engineering of Ministry of Education, School of Life Sciences, Jilin University, 2699 Qianjin Street, Changchun, 130012, P. R. China. Tel: +86-431-85155249; Fax +86-431-85155200

**^†^ These authors contributed equally to this work**

*** Corresponding author**

E-mail addresses: wanyouzhong@jlu.edu.cn (Y. Z. Wan); yudahai@jlu.edu.cn (D. H. Yu)

**Supplementary figures**


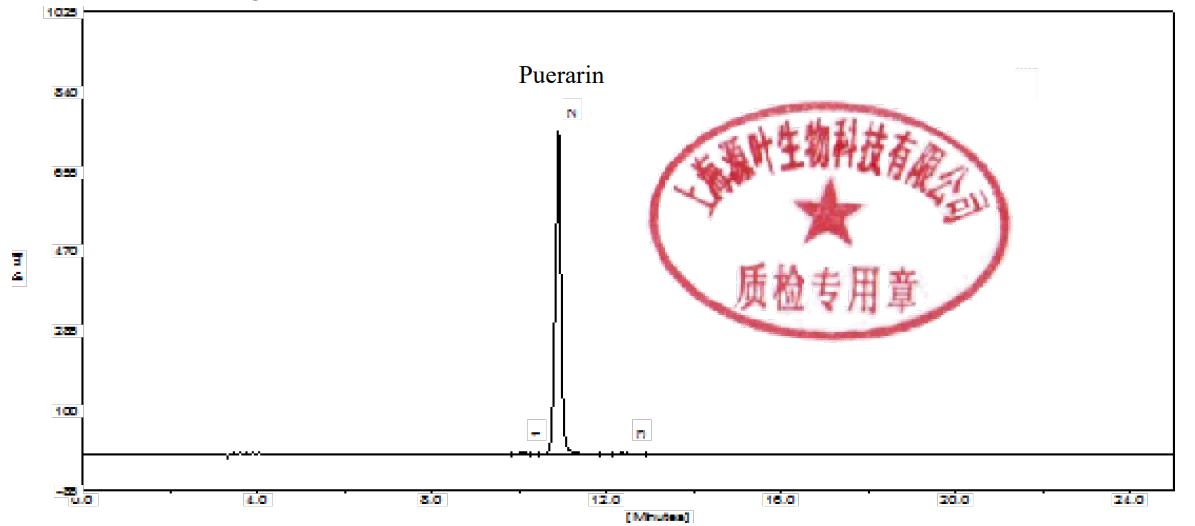


**FIGURE S1**

The chromatogram of the puerarin.


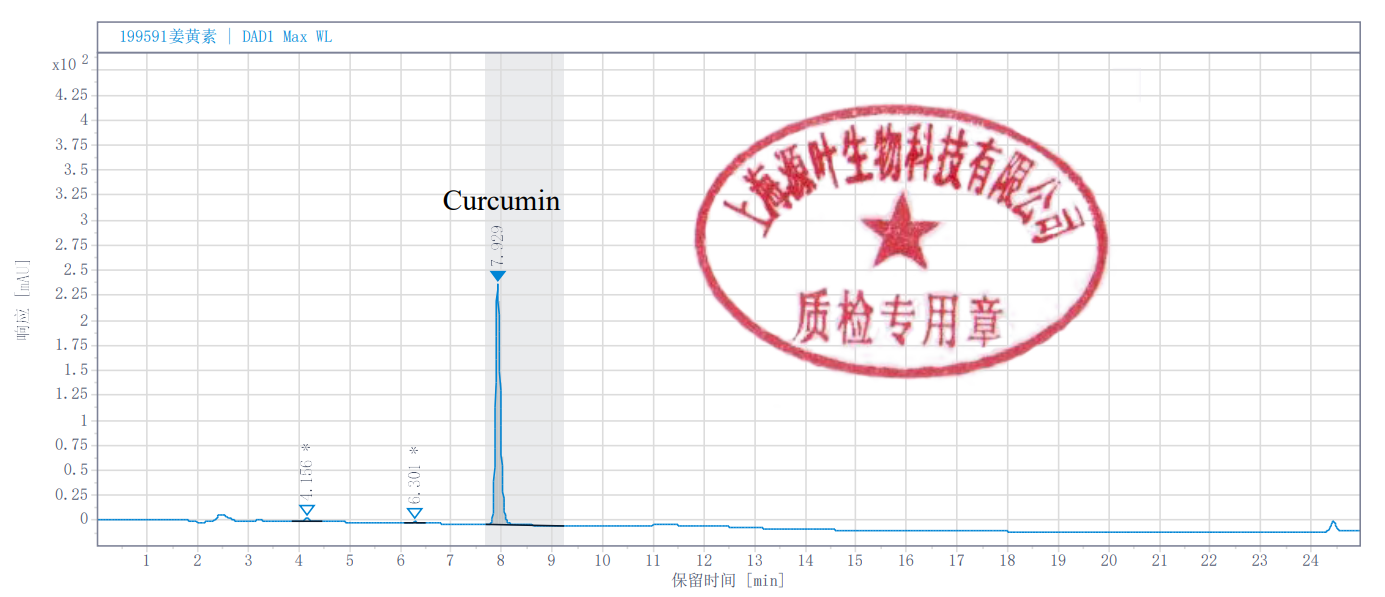


**FIGURE S2**

The chromatogram of the curcumin.


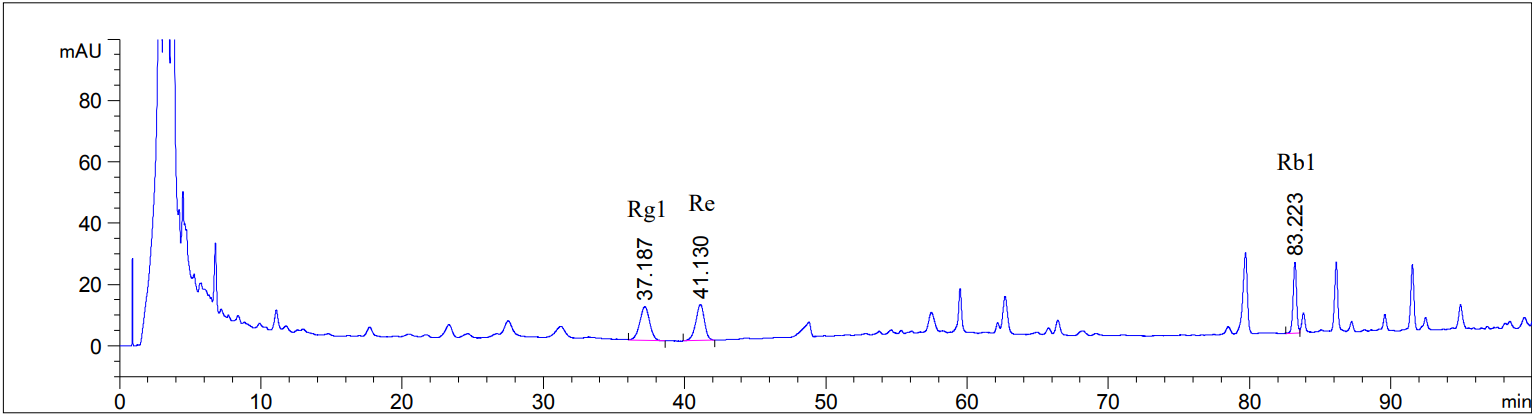


**FIGURE S3**

The chromatogram of the total ginsenosides.
